# Rhinovirus stimulated IFN-α production: how important are plasmacytoid DCs, monocytes and endosomal pH?

**DOI:** 10.1038/cti.2015.27

**Published:** 2015-10-30

**Authors:** Yang Xi, Arvid Finlayson, Oliva J White, Melanie L Carroll, John W Upham

**Affiliations:** 1 Lung and Allergy Research Centre, School of Medicine, The University of Queensland, Translational Research Institute (TRI), Woolloongabba, Queensland, Australia; 2 Department of Respiratory Medicine, Princess Alexandra Hospital, Woolloongabba, Queensland, Australia

## Abstract

Human rhinovirus (HRV) infection is a major cause of asthma exacerbations, which appears to be linked to a defective innate immune response to infection. Although the type I interferons (IFN-α and IFN-β) have a critical role in protecting against most viral infections, the cells responsible for IFN production in response to HRV and the relative importance of pattern recognition receptors located in endosomes has not been fully elucidated. In the current study we demonstrate that, using intracellular flow cytometry, >90% of the IFN-α-producing cells in human blood mononuclear cells following HRV16 exposure are plasmacytoid dendritic cells, whereas monocytes and myeloid dendritic cells contribute only 10% and <1%, respectively, of the IFN-α production. Bafilomycin and chloroquine, agents that inhibit the function of endosomal toll-like receptors (TLRs), significantly reduced the capacity of TLR3-, TLR7- and TLR-9-stimulated cells to produce IFN-α and the IFN-induced chemokine CXCL10 (IP-10). In contrast, only bafilomycin (but not chloroquine) effectively suppressed HRV16-stimulated IFN-α and IP-10 production, whereas neither bafilomycin or chloroquine inhibited HRV16-stimulated interleukin-6 release. Attempts to block IFN-α production with commercially available TLR-specific oligonucleotides were unsuccessful due to major ‘off-target' effects. These findings suggest that among circulating haemopoietic cells, plasmacytoid dendritic cells and TLRs located within endosomes are critical for inducing efficient IFN-I production in response to HRVs.

Human rhinovirus (HRV), a positive-sense, single-stranded RNA (ssRNA) virus, is recognized as the most common cause of viral upper respiratory tract infections. HRV is responsible for more than one-half of cold-like illnesses in healthy individuals.^1, 2^ In children and adults with asthma, HRV infections can have more serious consequences, being responsible for over 70% of acute asthma exacerbations leading to hospitalization.^3^ This vulnerability to HRV infections in people with asthma has been attributed to a deficient antiviral innate immune response involving both airway epithelial cells and migratory leukocyte populations.^4, 5, 6, 7^ According to this paradigm, deficient production of interferon (IFN)-α, IFN-β and IFN-λ is thought to facilitate HRV spread to the lower respiratory tract and a higher viral load.^4, 6, 8^ In contrast, other investigators have suggested that viral loads are similar in asthmatic and control subjects during HRV infections,^9^ and that asthma induces an unbalanced adaptive immune response, thereby leading to severe and longer-lasting airway inflammation.^10, 11^ HRV has also been shown to be the most common cause of lower respiratory tract infections in hematopoietic stem cell and lung transplant recipients, and is associated with a high risk of both acute and chronic rejection and subsequent higher mortality.^12, 13^

Although HRVs are extremely common in the community, many aspects of the innate immune response to infection remain unclear. Much is known about the way in which airway epithelial cells respond to HRV.^4, 6^ However, there are several unanswered questions regarding the way in which circulating, bone marrow-derived leukocyte populations respond to HRV. It is not clear which particular cells are most responsible for the HRV-induced type I IFN production, and the relative importance of individual pattern recognition receptors has not been elucidated. Early studies focussed on the capacity of HRVs to activate monocytes;^14^ however, our recent findings suggest that plasmacytoid dendritic cells (pDCs) are responsible for the majority of IFN-α and IFN-β synthesis. When peripheral blood mononuclear cells (PBMCs) were depleted of pDC via immune-magnetic beads, HRV-induced IFN-α production was reduced by 98%, relative to intact PBMC.^15^ However, it was not clear from this study whether pDCs themselves were producing IFN-α, or possibly acting indirectly via monocytes or other cells to induce IFN-α release.

In structural cells such as epithelial cells, HRVs replicate within cells and both ssRNA and double-stranded RNA are recognized by endosomal toll-like receptor 3 (TLR3) and the cytoplasmic receptor melanoma differentiation-associated gene 5.^16^ In contrast, ssRNA viruses do not generally replicate inside pDC, although this has not been examined specifically in relation to HRV.^14^ In pDCs, recognition of nucleic acids is largely dependent on endosomal receptors; this then induces IFN-I production via a MyD88- and IRF7-dependent pathway.^17, 18^

Therefore, the aims of this current study were first to examine the cellular source of IFN-α production in HRV-stimulated PBMCs using intracellular cytokine staining in combination with surface markers specific for pDCs, myeloid dendritic cells (mDCs) and monocytes. Second, we aimed to investigate the importance of endosomal TLRs using general inhibitors of endosomal function (bafilomycin and chloroquine), and inhibitory oligonucleotides (ODNs) directed against specific TLRs.

## Results

### HRV-16 (RV16)-stimulated IFN-α is mainly localized in pDC

To directly demonstrate the cells responsible for RV16-induced IFN-α, PBMCs from healthy subjects (*n*=9) were stimulated with RV16 for 24 h and the frequency of IFN-α-producing cells including monocytes (CD14^+^CD303^−^), mDCs (CD1c^+^) and pDCs (CD303^+^CD14^−^) was evaluated using flow cytometry.

**Figure 1:**
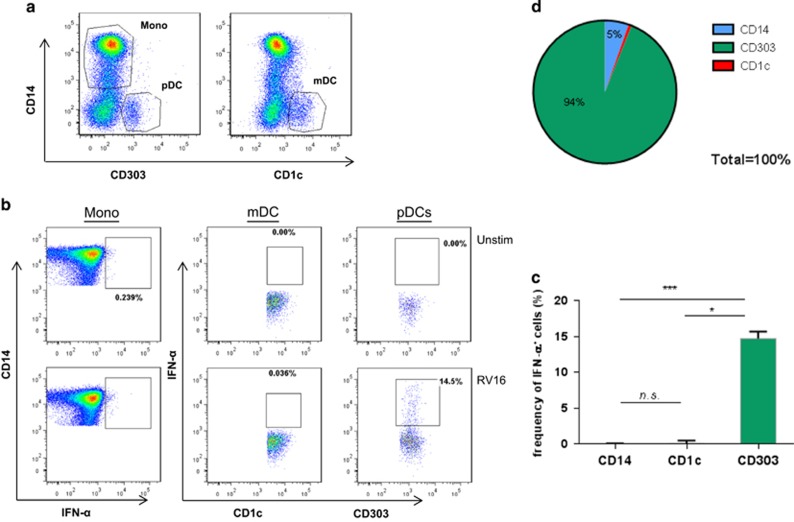
RV16 stimulation triggers significantly more IFN-α^+^CD303^+^ than IFN-α^+^CD14^+^ or IFN-α^+^CD1c^+^. PBMCs from healthy donors (*n*=9) were cultured in the presence of RV16 (multiplicity of infection=1) or media only (unstimulated control) for 24 h at 37 °C. Using intracellular cytokine staining, the percentage and/or proportion of IFN-α-producing cells in human PBMCs were evaluated. After gating on total live cells, monocytes (CD14^+^CD303^−^), mDCs (CD1c^+^CD14^−^) and pDCs (CD303^+^CD14^−^) were identified (**a**). Results for IFN-α-producing cells (**b**—from left to right, monocytes, mDCs or pDCs) are shown. The bar chart (**c**) shows the frequency of IFN-α-producing cells, and the plotted values represent the RV16-stimulated cells (**b**—bottom panels) minus the unstimulated cells (**b**—top panels). The proportion of IFN-α-producing cell was also evaluated (**d**). All data represent mean±s.e.m. *P<0.05, ***P<0.001. mono, monocytes; mDC, myeloid dendritic cell; pDC, plasmacytoid dendritic cell; IFN, interferon; RV16, rhinovirus-16.

Representative fluorescence-activated cell sorting plots indicate that both monocytes and pDCs exhibit elevated intracellular IFN-α following RV16 stimulation compared with their respective controls. In contrast, intracellular IFN-α could not be detected in mDC (Figures 1a and c). Pooled data from all donors show a considerably greater frequency of IFN-α^+^CD303^+^ pDC than IFN-α^+^CD14^+^ monocytes (*P<0.05) or IFN-α^+^CD1c^+^ mDC (***P<0.001; Figure 1c). Out of the three cell types tested, 94% of the IFN-α-producing cells were pDCs compared with that of the monocytes (5% of IFN-α production) or mDCs (<1% of IFN-α production; Figure 1d).

### How important are endosomal TLRs for the induction of IFN-α synthesis?

The capacity of endosomal TLRs (TLR3, TLR7 and TLR9) to recognize viral nucleic acids and to induce *IFN* gene expression appears to be pH dependent. Accordingly, the next set of experiments utilized bafilomycin and chloroquine, two well-characterized inhibitors of endosomal acidification.^19, 20^

**Figure 2:**
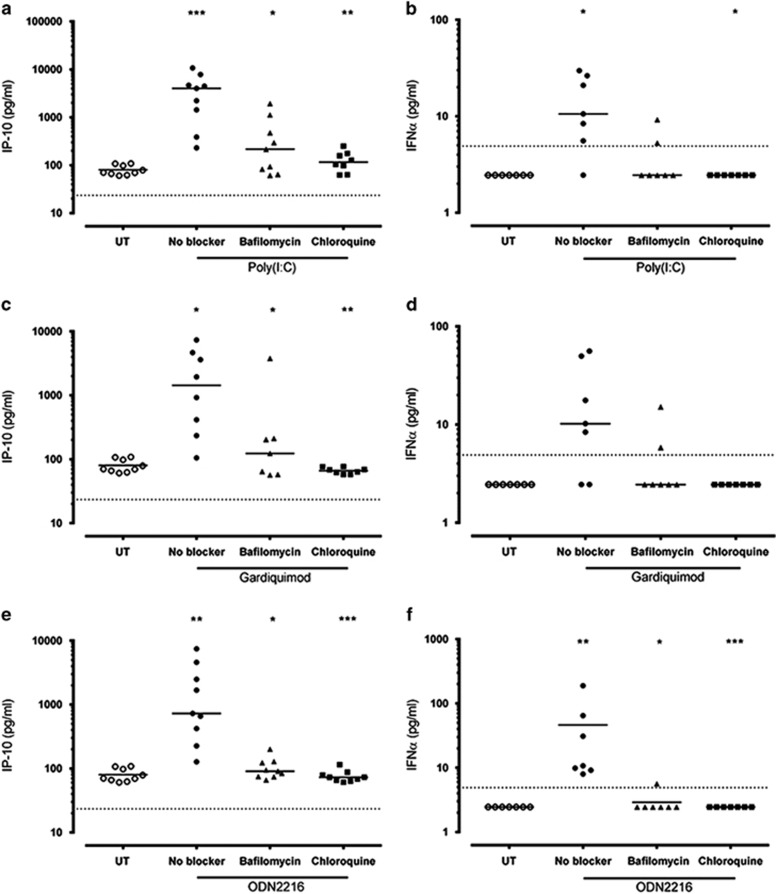
Bafilomycin and chloroquine effectively inhibit TLR3-, TLR7- and TLR9-specific IP-10 and IFN-α. PBMCs from healthy donors (*n*=7–9) were treated with 50 μM bafilomycin, 6 μM chloroquine or untreated, and subsequently stimulated with 25 μg ml^−1^ Poly (I:C) (**a**, **b**), 0.3 μg mml^−1^ gardiquimod (**c**, **d**), 50 μM ODN2216 (**e**, **f**) for 24 h at 37 °C. IP-10 protein (pg ml^−1^; left panels) and IFN-α protein (pg ml^−1^; right panels) were measured by ELISA. Solid lines represent the median for each condition. Dotted lines represent the limit of detection (23.4 pg ml^−1^ for IP-10 and 4.9 pg ml^−1^ for IFN-α). *P<0.05, **P<0.01, ***P<0.001 compared with TLR ligand-only treated condition. IFN, interferon; IP-10, interferon gamma-induced protein 10; TLR, toll-like receptor; UT, untreated.

PBMCs from the healthy subjects (*n*=7–9) were pretreated with 50 nm bafilomycin, 6 μm chloroquine or untreated, and subsequently stimulated with TLR3-, TLR7- and TLR9-specific agonists (Poly (I:C), Gardiquimod (Gq) and ODN2216). TLR3 and TLR9 stimulation resulted in significantly higher (*P*<0.05) production of IFN-α and the IFN-inducible chemokine CXCL10 (IP-10) than in untreated cells (Figures 2a, b and e–f). TLR7 ligand stimulation resulted in significantly increased (*P*<0.05) IP-10 production, with a non-significant trend for increased IFN-α production (Figures 2c and d). Both bafilomycin and chloroquine significantly inhibited (*P*<0.05) IP-10 production compared with TLR3, TLR7 and TLR9 ligand-only treated cells (Figures 2a, c and e). Both bafilomycin and chloroquine inhibited IFN-α production compared with ligand-only treated cells; some of these reductions were statistically significant (Figures 2b, d and f).

### Non-specific effects of ODNs

**Figure 3:**
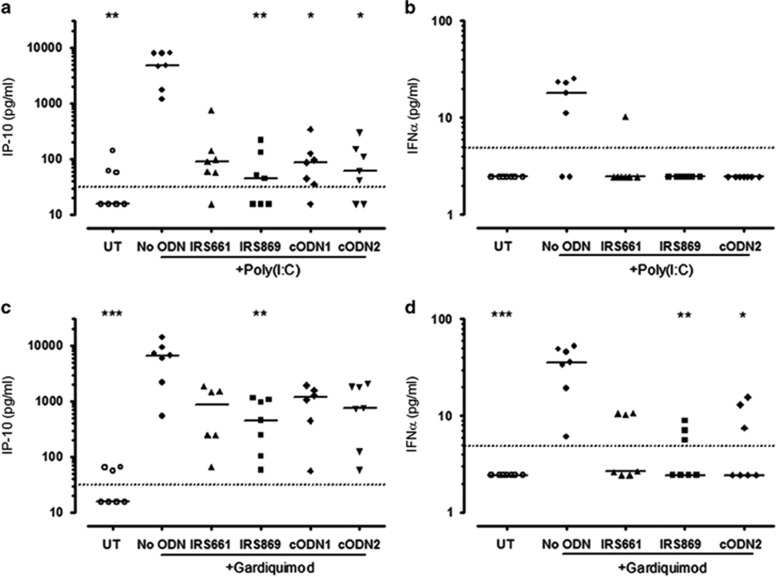
PS ODNs inhibit TLR3 and TLR7. PBMCs from healthy donors (*n*=6–7) were treated with 1.4 μm IRS661, 2.8 μm IRS869, 2.8 μm cODN1, 2.8 μm cODN2 or untreated and then stimulated with 25 μg ml^−1^ Poly (I:C) (TLR3; **a**, **b**) or 0.3 μg ml^−1^ gardiquimod (TLR7; **c**, **d**) or left unstimulated for 24 h at 37 °C. Solid lines represent medians and dotted lines represent limit of detection. *P<0.05, **P<0.01, ***P<0.001 compared with TLR ligand-only treated condition. cODN, control ODN; IFN, interferon; IP-10, interferon gamma-induced protein 10; ODN, oligonucleotide.

We next investigated the inhibition efficiency and specificity of the individual TLR-specific ODNs, IRS661, IRS869 and two control ODNs (cODN1 and cODN2). PBMCs from healthy individuals were cultured with 1.4 μm of IRS661 or 2.8 μm IRS869, cODN1 or cODN2 and then stimulated with Gq or Poly (I:C). Somewhat unexpectedly, many of these ODNs exhibited non-specific inhibition of IP-10 and IFN-α production (Figure 3). For example, IRS661 (supposedly a TLR7 antagonist) inhibited TLR3-stimulated IP-10 production (Figure 3a). Similarly, IRS869 (supposedly a TLR9 antagonist) inhibited TLR3- and TLR7-stimulated IP-10 production (Figures 3a and c). The two cODNs, cODN1 and cODN2, unexpectedly inhibited TLR3-stimulated IP-10 production (Figure 3a). Similar non-specific effects were also seen in relation to IFN-α production (Figure 3b).

### Bafilomycin inhibits RV16-stimulated innate immune responses

**Figure 4:**
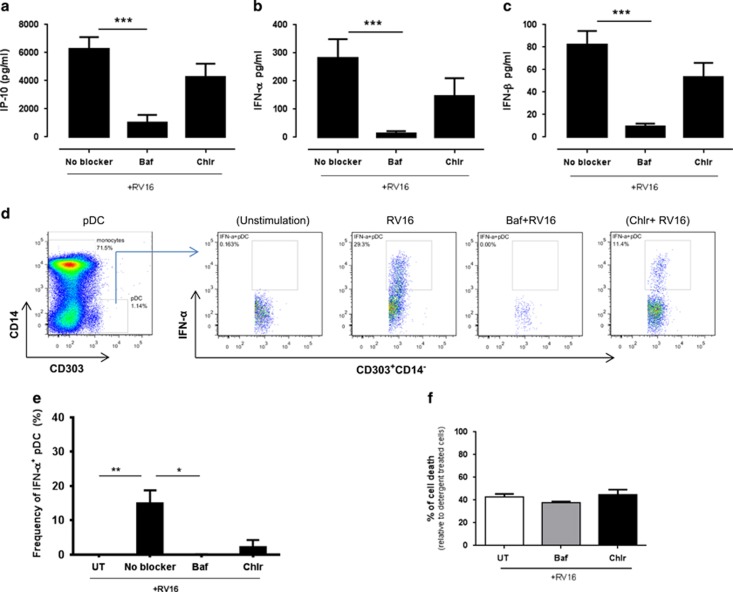
Bafilomycin, but not chloroquine, significantly inhibits RV16-stimulated IP-10, IFN-α/β synthesis in health. PBMCs from healthy donors were treated with 50 nm bafilomycin, 6 μm chloroquine or left as untreated, and subsequently stimulated with RV16 for 24 h at 37 °C. IP-10, IFN-α and IFN-β protein were measured using ELISA (**a–c**, *n*=10). Data represent mean values where after the unstimulated control was subtracted. The frequency of the IFN-α-producing cell was evaluated using intracellular cytokine staining and the applied gating strategy was the same as in Figure 1. The IFN-α-producing pDC in the variable treatments was then evaluated (**d** from left to right, unstimulated control, RV16 stimulation only, RV16 stimulation of Baf-treated cells and RV16 stimulation of Chlr-treated cells). The cytotoxicity of bafilomycin and chloroquine was evaluated (*n*=5) using LDH Cytotoxicity Assay (**f**). All data represent mean±s.e.m. *P<0.05, **P<0.01, ***P<0.001. Baf, bafilomycin; Chlr, chloroquine; IFN, interferon; LDH, lactate dehydrogenase; No blocker, media only; RV16, rhinovirus-16; UT, untreated.

As bafilomycin and chloroquine effectively inhibited TLR ligand-induced IP-10 and IFN-α, we investigated whether these inhibitors also inhibit RV16-stimulated responses. PBMCs from healthy subjects were pretreated with bafilomycin, chloroquine or untreated, and subsequently stimulated with RV16. Similar to what was observed following TLR ligand stimulation, bafilomycin inhibited RV16-stimulated IP-10, IFN-α and IFN-β production (Figures 4a and c) and markedly reduced the frequency of IFN-α^+^-producing pDCs (Figures 4 d–e). In contrast, chloroquine did not have consistent effects on RV16-stimulated IP-10, IFN-α and IFN-β production, and the changes in cytokine release were not statistically significant (Figures 4 a–c). It is noteworthy that neither bafilomycin nor chloroquine inhibited HRV16-stimulated interleukin-6 release (Supplementary Figure S1). Bafilomycin and chloroquine did not appear to be cytotoxic to the cells, as measurement of lactate dehydrogenase (LDH) in the supernatants showed that bafilomycin and chloroquine did not increase cell death over and above that seen in HRV16-stimulated cells (Figure 4f).

### Bafilomycin also inhibits RV16-stimulated innate immune responses in asthma

**Figure 5:**
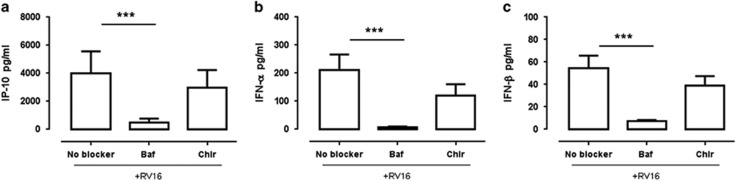
Bafilomycin significantly inhibits RV16-stimulated IP-10 and IFN-α/β in asthma. PBMCs from asthmatic subjects (*n*=10) were treated with media alone, bafilomycin or chloroquine, and then stimulated with RV16 for 24 h at 37 °C. IP-10 protein (**a**), IFN-α (**b**) and IFN-β (**c**) were measured by ELISA. Data represent mean values where the unstimulated control was subtracted, error bars represent s.e.m. ***P<0.001.

**Figure 6:**
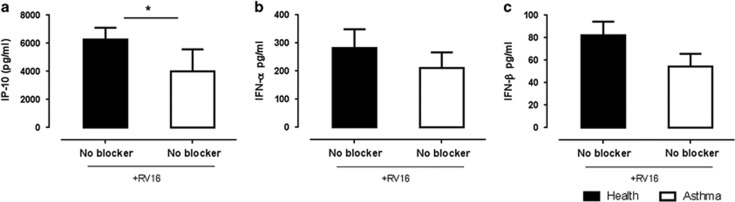
RV16-induced IP-10 but not IFN-I (α/β) is abrogated in asthma compared healthy controls. PBMCs from healthy (*n*=10; solid bars) and asthmatic (*n*=10; open bars) subjects were stimulated with RV16 or media alone for 24 h at 37 °C. IP-10 protein (**a**), IFN-α (**b**) and IFN-β (**c**) were measured by ELISA. Data represent average values where the unstimulated control was subtracted, error bars represent s.e.m. *P<0.05.

Similar to what was observed in the healthy subjects, bafilomycin, but not chloroquine, significantly inhibited RV16-stimulated IP-10, IFN-α and IFN-β in PBMCs from asthmatic donors (Figures 5a and c). Interestingly, we have found that cells from the asthmatic subjects produced significantly lower IP-10 than healthy subjects, whereas IFN-α and IFN-β production were similar in asthmatic and healthy subjects (Figures 6a and c).

## Discussion

Even though HRV infections are extremely common in the community, and can cause severe morbidity in those with pre-existing chronic lung disease, many aspects of the innate immune response to HRV remain unclear. Much progress has been made in dissecting airway epithelial responses to HRV, identifying the pattern recognition receptors and intracellular signaling pathways that lead to IFN-I and IFN-III production and the induction of an antiviral state.^21, 22^ However, much less is known about migratory bone marrow-derived leukocyte populations and how these respond to HRV.

The key findings to emerge from this study are that pDCs and TLRs located within endosomes have a major role in the innate immune response to HRV, recognizing viral nucleic acids and inducing efficient production of IFN-I and downstream molecules such as IP-10 (CXCL10).

Although pDCs have long been regarded as the ‘natural type I IFN-producing cell', dedicating 60% or more of their messenger RNA to IFN production,^23^ the role that pDCs have in IFN production in HRV infections has not been clear. Our previous study has shown that the IFN-α/β productions are markedly diminished when pDCs are depleted from PBMCs.^15^ In the current study, we have confirmed using intracellular cytokine staining that RV16-stimulated IFN-α production by circulating cells is largely localized to pDCs, and to a lesser extent monocytes. In contrast, mDCs do not contribute to IFN-I synthesis, at least not within the first 24 h.

As antigen-presenting cells, pDCs can rapidly recognize viral nucleic acids to produce antiviral IFN-I and inhibit viral replication.^24^ We used a variety of approaches in an attempt to determine which subcellular compartments and which pattern recognition receptors are most important for recognition of HRV. Using two inhibitors of endosomal acidification, bafilomycin and chloroquine, we observed reductions in TLR3, TLR7 and TLR9 signaling as shown in Figure 2, similar to findings reported by others.^19^ We also tested two commercially available ODNs with the aim of specifically inhibiting TLR7 and TLR9. However, both IRS661 and IRS869 showed lack of specificity in their ability to inhibit TLR7 or TLR9 signaling in our model (Figure 3); even the cODNs showed inhibition of some responses. Our findings contrast with those of Barrat *et al.* who initially reported that IRS661 and IRS869 specifically inhibit TLR7- and TLR9-stimulated cytokine production by human pDCs.^25^ Although some studies show that ODNs lead to TLR inhibition, none of them have directly demonstrated the sequence specificity of the ODNs tested. Our findings, and those reported by others, suggested that phosphorothioate (PS) ODNs can have ‘off-target' effects, suggesting that TLR7 inhibition by PS ODN is not sequence specific.^26, 27, 28^ This lack of ODN specificity makes it difficult to use these ODNs to accurately define the importance of TLR7 and TLR9 signaling in our system. It is hoped that further refinements to the design of ODNs in the future will improve specificity available for testing in future.

In relation to the HRV-stimulated cultures, we found that bafilomycin significantly inhibited IFN-α, IFN-β and IP-10 production in healthy donors (Figure 4). In contrast, there was a trend for chloroquine to inhibit IP-10, IFN-α and IFN-β production, but this was not statistically significant. Although there is some evidence that bafilomycin can alter viral uncoating and entry into cells, the fact that bafilomycin inhibited type I IFN release, but not interleukin-6 release, makes it likely that the effects of bafilomycin observed herein are related to effects on endosomal function, rather than non-specific inhibition of viral entry into cells. It was important to demonstrate that the inhibitory effects of bafilomycin on innate immune function were not due to cytotoxicity. Measurement of LDH activity in culture supernatant suggests that this was not the case, although it is difficult to exclude a minor degree of cytotoxicity associated with the combination of chloroquine and RV16.

Although bafilomycin and chloroquine both inhibit endosomal function, the mechanisms involved appear to be distinct. Bafilomycin is a specific inhibitor of vacuolar-type H^+^-ATPase that prevents acidification of endosomes, and inhibits the activity of pH dependent lysosomal proteases.^19, 29, 30^ Recent data suggest that chloroquine acts by inducing conformational modifications in nucleic acids that reduce TLR binding, rather than by changing endosomal pH.^19^ Our findings highlight endosomal pH, and TLRs located within endosomes, as important factors involved in the initiation of IFN-I synthesis during HRV infections in healthy people and those with asthma. TLR3, TLR7, TLR8 and TLR9 are all located within endosomes: in the setting of HRV, we believe that TLR7 within pDC is likely to be most important of these TLRs given its capacity to recognize ssRNA. Formal proof of this notion will require the development of more specific TLR inhibitors. Although TLR3 is critical for recognizing dsRNA produced during viral replication, our previous work indicates that HRV does not replicate within PBMC.^15^ Our findings do not preclude the involvement of cytosolic pattern recognition receptors such as RIG-I and melanoma differentiation-associated gene 5, although we would argue there will be minimal free HRV in the cytoplasm of pDC in the absence of viral replication.

Although a detailed comparison of healthy donors and those with asthma was not the primary focus of this study, a number of important observations are worth discussing. PBMCs from asthmatics exhibited lower RV16-stimulated IP-10 production than non-asthmatics. Prior studies have shown that infection of bronchial epithelial cells from people with asthma results in decreased IFN-I and IFN-III production compared with epithelial cells from non-asthmatic subjects.^4, 6^ In addition, PBMCs infected with respiratory syncytial virus and Newcastle disease virus, both ssRNA viruses, also showed decreased expression of IFN-α in people with asthma compared with healthy subjects.^31, 32^ However, there was no difference in the production of IFN-α or IFN-β in PBMCs following RV16 stimulation in the current study, possibly due to the fact that most asthmatic participants in this study had relatively mild- and well-controlled disease. Sykes *et al.*^33^ have also recently reported that HRV-induced IFN production is not deficient in well-controlled asthma. Whether the low IP-10 production in the current study might be related to deficiencies in other type of IFN (for example, IFN-λ) warrants further investigated as there is evidence that IFN-λ can induce IP-10 production.^34^ Bafilomycin inhibited HRV-induced IFN-α, IFN-β and IP-10 production in those with asthma (Figure 6), similar to the results seen in healthy subjects (Figure 4), suggesting that there is no gross deficit in endosomal function in asthma. Whether endosomal function is specifically altered in more severe asthmatics is an issue that warrants future study. Some investigators have reported that genetic variations in *TLR7* and reduced TLR7 expression and function are associated with asthma,^35, 36, 37, 38^ Given that TLR7 is most strongly expressed in pDCs and our current finding that the pDCs are the primary cell responsible for RV16-stimulated IFN production will be important for future studies of antiviral immunity in asthma to focus on pDC and TLR7.

In summary, we have demonstrated that RV16-stimulated IFN-α secretion in human PBMCs is mainly localized in pDCs. The IFN-α response in pDCs is suppressed by addition of bafilomycin suggesting the involvement of endosomal TLRs. Compared the healthy subjects, the asthmatic patients produced lower IP-10.

## Methods

### Patients

Healthy adult volunteers or asthmatic patients were recruited from Respiratory and Immunology Clinics at the Princess Alexandra Hospital, Woolloongabba, Queensland. The study was approved by the Metro South Human Research Ethics Committee, and all subjects provided written informed consent.

### Reagents and viruses

Inhibitory and non-inhibitory cODNs (Geneworks, Thebarton, SA, Australia) were synthesized with a PS backbone and ODN 2261 (TLR9 agonist; Invivogen, San Diego, CA, USA), a type A CpG ODN, was synthesized with a mixed PS and PO backbone. All ODN sequences were derived from previous studies.^25, 39, 40^ ODNs were reconstituted in sterile phosphate-buffered saline and then stored at −20 °C. The ODN sequences can be found in Table 1. Chloroquine diphosphate salt (Sigma-Aldrich, St Louis, MO, USA) was reconstituted in sterile phosphate-buffered saline and then stored at 4 °C for no >4 weeks. Bafilomycin (Sigma-Aldrich) was reconstituted in sterile dimethylsulfoxide and stored at −20 °C. Gardiquimod (Gq; TLR7/8 agonist; Invivogen) and Polyinosinic:polycytidylic acid (Poly (I:C); TLR3 agonist; Invivogen) were reconstituted in endotoxin-free water and stored at −20 °C. Following initial dose response experiments, TLR agonists were used at the following concentrations: 0.3 μg ml^−1^ Gq, 0.1 μg ml^−1^ ODN2216 or 25 μg ml^−1^ Poly (I:C). TLR antagonists/inhibitors were used as following concentrations: 1.4 μm IRS661 or 2.8 μm IRS869, cODN1, cODN2, 50 nm bafilomycin and 6um chloroquine.

RV16 stocks were generated by passage in Ohio HeLa cells as described previously by Sanders *et al.*^41^ followed by purification over an OptiPrep gradient (Sigma-Aldrich). To define the optimal concentration of RV16, the 50% tissue culture-infective dose was determined as previously described by Pritchard *et al.*^15^

### Cell separation and culture

Cryopreserved PBMCs were thawed rapidly in a 37-°C water bath and resuspended drop wise in 10 ml of cold Roswell Park Memorial Institute 1640 media containing 2% heat-inactivated fetal calf serum. Cell suspensions were prepared in Roswell Park Memorial Institute 1640 supplemented with antibiotics, 2-ME, and 10% heat-inactivated fetal calf serum (24 h, innate immune studies). Cells (2 × 10^6^ or 1 × 10^6^) per well were seeded in a 96-well U-bottom plate in the presence of RV16 at a multiplicity of infection of 1 or with media only. Cells were incubated at 37 °C with 5% CO_2_ incubator with 95% humidity for 24 h.

For the functional study of endosomal TLRs, the cells were pretreated with endosomal TLR inhibitors, PS-linked ODN or medium alone at 37 °C with 5% CO_2_ for 1 h, and subsequently stimulated with TLR agonists or RV16 (multiplicity of infection of 1) in the same condition for 24 h. Following culture, plates were centrifuged at 750 *g* for 5 min and the supernatants were collected and stored at −20 °C for enzyme-linked immunosorbent assay (ELISA) analysis or LDH activity analysis.

### Intracellular cytokine analysis

Intracellular cytokine staining was used to assess IFN-α-producing pDCs, monocytes and mDCs after RV16 (multiplicity of infection of 1) stimulation. PBMCs (2 × 10^6^) per well were seeded in a 96-well U-bottom plate and stimulated with or without RV16 at 37 °C with 5% CO_2_ for 18 h, and further incubated with Brefeldin A (eBioscience, San Diego, CA, USA) for 4 h. Cells were washed with FACs buffer (1% heat-inactivated fetal calf serum in phosphate-buffered saline; fetal calf serum; Bovogen Biologicals, Keilor East, VIC, Australia) and incubated with normal goat IgG (Sigma-Aldrich) at 4 °C for 15 min to block non-specific Fc binding. The cells then were surface stained with CD303-PE, CD14-PerCP and CD1c-FITC (Miltenyi Biotec, Bergisch Gladbach, Germany) for 30 min at 4 °C, then fixed and permeabilized before antigen-presenting cell conjugated anti-IFN-α (Miltenyi Biotec) intracellular staining for 30 min at 4 °C. The cells were then washed twice with the FACs buffer, finally fixed in 0.5% paraformaldehyde before analysis. A total of ~500 000 gated events per sample were collected using FACS Canto (BD Biosciences, Franklin Lakes, NJ, USA), and the results were analyzed using the FlowJo Tree Star software (version 7.6.1; Flowjo LLC, Ashland, OR, USA). Unstimulated background was subtracted from the data.

### Cytokine ELISA

Cell culture supernatants collected were assayed by ELISA to measure the concentration of cytokines. IP-10 (also known as CXCL10) was measured using commercially available paired Abs and recombinant cytokines (BD Biosciences). The ELISAs were performed using a standard protocol optimized by our group.^42^ IFN-α and IFN-β ELISA were, respectively, performed using VeriKineTM Human IFN alpha serum Sample ELISA kit (PBL Assay Science, Product#41110, Picastaway, NJ, USA) or Human IFN-β Elisa Kit (elisakit.com, product#0041, Scoresby, VIC, Australia), according to the manufacturer's instructions.

### LDH cytotoxicity assay

Cells (0.5 × 10^6^) per well were pretreated with (1) Roswell Park Memorial Institute media alone, (2) bafilomycin or (3) chloroquine for 45 min, and stimulated with/without RV16 (multiplicity of infection of 1) for further 23 h at 37 °C. Cell culture supernatant was collected and the percentage of the cell death relative to detergent-treated sample was measured using LDH Cytotoxicity Assay kit (Cat#1780; Promega, Madison, WI, USA) according to the manufacturer's instruction. The percentage of cytotoxicity is calculated as:

Absorbance of each sample/Abs maximum lysate control × 100%.

### Statistical analysis

Data were analyzed in IBM SPSS Statistics and GraphPad Prism 5 (GraphPad Software, San Diego, CA, USA) using Friedman tests with Dunn's post-tests to compare paired samples, whereas Mann–Whitney test was used to compare data from asthmatic and healthy subjects. Raw data are presented as mean±s.e.m. The Figures 2 and 3 represent the median and interquartile ranges. The *P-*values <0.05 were considered significant.

## Figures and Tables

**Table 1:** Name and sequence of oligonucleotides

*Name*	*Sequence (5′-3′)*
ODN IRS661	TGCTTGCAAGCTTGCAAGCA
ODN IRS869	TCCTGGAGGGGTTGT
cODN1	TCCTGCAGGTTAAGT
cODN2	TCCTGGCGGAAAAGT
ODN 2261	GGggacgatcgtcGGGGGG^a^

a Lower case letters indicate phosphodiester linkage, whereas upper case letters indicate phorothioate linkage.

## Notes

The authors declared no conflict of interest.
