# Supplementary material for: Rhinovirus stimulated IFN-α production: how important are plasmacytoid DCs, monocytes and endosomal pH?
Source: Clin Transl Immunology. 2015 Oct 30;4(10):e46–. doi: 10.1038/cti.2015.27 (PMC4673444; doi:10.1038/cti.2015.27)
Supplement: Supplementary Information [file cti201527x1.pdf]

Figure-S1

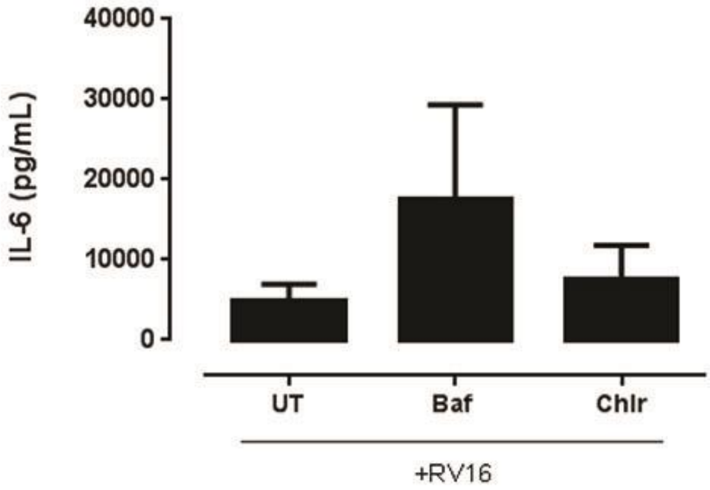

Figure-S1 *Effects of bafilomycin and chloroquine on IL-6 production in healthy subjects.* PBMC from asthmatic subjects (n=10) were treated with bafilomycin, chloroquine or media alone, and then stimulated with RV16 for 24 hrs at 37°C. IL-6 protein was measured by ELISA. Data represent mean values where the unstimulated control was subtracted.
